# Assessing the quality of life of health-referred children and adolescents with short stature: development and psychometric testing of the QoLISSY instrument

**DOI:** 10.1186/1477-7525-11-76

**Published:** 2013-05-07

**Authors:** Monika Bullinger, Julia Quitmann, Mick Power, Michael Herdman, Emmanuelle Mimoun, Kendra DeBusk, Eva Feigerlova, Carolina Lunde, Maria Dellenmark-Blom, Dolores Sanz, Anja Rohenkohl, Andreas Pleil, Hartmut Wollmann, John E Chaplin

**Affiliations:** 1 University Hamburg-Eppendorf, Department of Medical Psychology, Martinistr. 52, W26, Hamburg 20246, Germany; 2 Department of Clinical Psychology, University of Edinburgh, Teviot Place, Edinburgh EH8 9AG, Scotland; 3 IMIM University, Insight Consulting and Research, Cami Ral 266, Mataró, Barcelona, Spain; 4 Department of Pediatric Endocrinology, University of Toulouse, CHU, 330 Avenue de Grande-Bretagne, Toulouse FR-31059, France; 5 Department of Paediatrics, Växthuset, Queen Silvia’s Children’s Hospital, Sahlgrenska Academy at University of Gothenburg, Gothenburg 41685, Sweden; 6 Pfizer, Inc. Specialty Care MDG, Outcomes Research, 10646 Science Center Drive, San Diego CA 92009, USA; 7 Pfizer Ltd., Specialty Care MDG, Endocrinology, Dorking Road, Walton-on-the-Hill Walton Oaks, Tadworth Surrey KT20 7NS, UK

**Keywords:** Health-related quality of life, Short stature, Growth hormone deficiency, Assessment in children, Idiopathic short stature, Outcomes research, Patient reported outcomes

## Abstract

**Background:**

When evaluating the outcomes of treatment in paediatric endocrinology, the health-related quality of life (HrQoL) of the child is to be taken into consideration. Since few self–reported HrQoL instruments exist for children with diagnosed short stature (dSS), the objective of this study was to develop and psychometrically test a targeted HrQoL instrument for use in multinational clinical research.

**Methods:**

The target population were short stature (height < −2 SDS) children and adolescents (age 8–12 and 13–18 years) with a diagnosis of growth hormone deficiency (GHD) or idiopathic short stature (ISS), differing in growth hormone treatment status. Focus group discussions for concept and item generation, piloting of the questionnaire with cognitive debriefing, and instrument field testing with a retest were conducted simultaneously in five countries. After qualitative and preliminary quantitative analyses, psychometric testing of field test data in terms of reliability and validity including confirmatory factor analyses (CFA) was performed.

**Results:**

Following item generation from focus group discussions, 124 items were included in a pilot test with a cognitive debriefing exercise providing preliminary feedback on item and domain operating characteristics. A field test with 268 participants showed high internal consistency reliabilities (alpha 0.82 – 0.95), good correlations with generic measures (up to r = .58), significant known group differences (e.g. in height: F = 32, df 244, p < 0.001) and an acceptable CFA model fit suggesting construct validity of the three-domain core structure with 22 items, supplemented by three mediator domains with 28 items.

**Conclusions:**

The QoLISSY questionnaire is a promising step forward in assessing the impact of dSS on HrQoL. It is based on items generated from the subjective experience of short stature children referred for endocrine investigation, is validated for use in five languages and it is easy to administer in clinical and research settings.

## Background

Health-related quality of life (HrQoL) is increasingly seen as an important outcome in clinical medicine. HrQoL includes subjective physical, emotional and social aspects of well-being and functioning from the patient’s perspective and extends to the perspective of other persons [1,2]. To understand the effects of a health condition on well-being and functioning in paediatric medicine, it is important to capture the view of the young patients and their parents. Very few instruments have been developed in the area of paediatric endocrinology, and assessments of well-being and functioning from the perspectives of children and adolescents are needed. Specifically, there is a lack of validated instruments to assess HrQoL in children and adolescents diagnosed with short stature (dSS) [3-6].

In two recent literature reviews, measures to assess HrQoL in dSS were identified; however, it was found that none were aimed at dSS as an isolated characteristic, only a few were self-reported and none specifically considered the HrQoL of dSS children with idiopathic short stature (ISS) [2,7]. Furthermore, most measures were developed for use within a single language population, so that pooling data across several countries could not be undertaken and comparison of the HrQoL impact of dSS across countries is not possible [8]. As a result of the paucity of instruments few studies have examined the effect of growth hormone (GH) treatment on HrQoL in short children and adolescents [9-14]. Stephen et al. [15] is one of the few papers which examined HrQoL in treated and untreated children with dSS using a generic instrument (PedsQL). The results suggest that some differences between dSS and normal height children exist, but the measure was not designed to include the specific problems of short stature children and therefore may be unable to detect differences between treated and untreated children.

The aim of the work presented here was to develop a methodologically sound condition specific HrQoL instrument for dSS, which is conceptually appropriate for a health-referral population, can be completed from both the child and parent perspectives and is available in several languages. This instrument was planned to be applicable in a wide range of research and clinical contexts, ranging from observational studies regarding the burden of dSS to randomized clinical trials examining treatment effects. In view of the need for a multi-language measure, instrument development and testing were performed simultaneously in five European countries (France, Germany, Spain, Sweden and the UK). Additional validation is on-going in other countries and languages.

In general, dSS is defined statistically in reference to the average height for a person's age, sex and is best expressed as a standard deviation score (SDS) away from the average for the comparison population. The definition of dSS therefore depends on representative population data which is collected on a national level. In general, a height SDS below −2 is considered to be short, may be an indication of an underlying, known or unknown, medical condition, and would be referred for assessment by an endocrine clinic. By using SDS in relation to parental heights instead of height in cm it is possible to make comparisons across genetic dispositions, age groups and gender [16,17]. Sex-specific reference data for height have been published for most developed countries and ethnic sub-populations thus allowing an auxological assessment of the child and the family to be used to identify dSS early in childhood.

Factors regulating somatic growth have been intensively researched and can be differentiated with regard to genetic, pathophysiological and environmental factors. Short stature may be associated with a number of health conditions such as prolonged hormone deficiency, systemic disease (e.g. chronic kidney disease, chronic inflammation), chromosomal abnormalities, inherited diseases, birth defect syndromes or may not be attributable to any specific cause (idiopathic short stature [ISS]) [18].

For children whose height is substantially below the norm for age and gender, short stature as an isolated characteristic may constitute a risk factor for behavioural and emotional problems. This is not only because of barriers in everyday life caused by height-related physical limitations, but also because short stature can be regarded as a social stigma, which in turn may affect self-perception and the social integration of persons with short stature. The intensity of impairment depends not only on the degree of short stature, but also on the way the individuals perceive their height and their ability to cope with the stigma [19-22].

Short stature, as a medical condition, can be ascertained early in life and depending on the underlying diagnosis it can be treated with GH therapy from an early age in childhood. Growth hormone deficiency (GHD) as one of the endocrine causes of short stature is the principle indicator for GH treatment [23]. However, although treatment with GH is also approved in other, non-GHD conditions where dSS is a symptom such as in Turner syndrome or ISS, regulations regarding which conditions are approved for GH treatment can differ between countries [24,25].

The most common growth related conditions presented at a growth clinic are GHD and ISS. The majority of children with identified GHD are treated with GH [26-28]. In contrast, although the clinical effectiveness of GH treatment in ISS is well documented [27,29,30] it is seldom treated even in countries where it is an approved condition [31]. In Europe, treatment of ISS with GH is not approved by the European Medicines Agency [25], whereas in the United States this indication has been approved since 2003.

The primary aim of GH treatment in children with dSS is to substitute the GH needed for normal development and to increase growth velocity as well as final height, with the expectation that this will also increase well-being and functioning [32]. An association between height and HrQoL has been documented in adults in the UK [33]. However, the treatment effect on children’s and adolescents’ HrQoL has not been similarly documented.

So far, studies have addressed the parent perspective on dSS more frequently than the perspective of the young patients themselves. Consequently more is known about how parents view the impact of dSS on their children, themselves, and their families [34-37].

The psychological aspects of dSS have been discussed in the literature for a number of years, starting with earlier publications about the impact of dSS on psychological functioning and the role of adaptation. However, past research has not explicitly considered the effect of dSS as an isolated characteristic in the broader context of well-being and HrQoL [38-42].

The current study aimed to develop an instrument to assess HrQoL in children and adolescents with short stature referred to a specialized growth center.

Since the main focus was on diagnosed short stature as an isolated characteristic, short children with other medical conditions potentially affecting HrQoL were excluded, as these conditions are frequently associated with dysmorphic features, pronounced comorbidities or developmental delays (such as in Turner syndrome, Prader-Willi syndrome, or in children born small for gestational age).

The main objective of this paper is to describe the operating characteristics and psychometric performance of the Quality of Life in Short Stature Youth (QoLISSY) measure in children and adolescents. The results of the development of the parent version will be reported separately as will be the detailed cross-cultural analysis of child and parent versions.

## Methods

The development of the QoLISSY instrument was carried out in three stages, conducted simultaneously in the five countries according to standardized guidelines, [43,44]:

•identification of relevant HrQoL concepts, dimensions and items in focus groups,

•pilot testing and cognitive debriefing of the preliminary questionnaire and

•analysis of psychometric properties of the instrument in a field test with a retest.

The study was reviewed and approved by the relevant ethics committees at the centers as required by national or regional regulation. All development stages were carried out in each participating country: France, Germany, Spain, Sweden and the United Kingdom.

In each of the collaborating countries HrQoL researchers worked with paediatric endocrinologists who recruited patients and parents for inclusion in the study. In order to capture the views of those concerned, the focus on short stature youth was limited to families involved in health care utilization. Based on medical records, patients between the ages of 8 and 18 who were (or had been before treatment) 2.0 standard deviations (SD) or more below the mean for height in that country because of GHD or ISS were eligible to participate. Children were recruited independent of treatment status; i.e. being treatment naïve, currently being treated or having had prior but not current treatment. Parents were also included in the study and respective results will be reported in a separate paper.

### Focus groups

Patient inclusion was conducted through endocrinology centers in the respective countries. Centers identified potential participant families based on clinic records and solicited their participation by mail which included information about the study along with a consent form. The composition of focus groups was determined by age (8–12, 13–18 years).

The focus group discussion was based on a semi-structured interview developed in prior international paediatric HrQoL research [45]. It was moderated by a trained interviewer and an assistant, who asked questions beginning with life in general and then focused on well-being and functioning with dSS. All discussions were audio-taped and transcribed into the local language.

The transcripts were screened by the researchers and relevant verbatim statements were abstracted, taking care to retain the wording used by the focus group subjects. Each statement was annotated with an identification of age, gender, diagnosis, treatment status and country. Analysis of the statements was carried out in a consensus group meeting of the project investigators. All statements were copied onto individual cards in both their original language and in a translation to English conducted by bilingual (local and English) study center personnel. The use of English as a core language reflects the need to have a mechanism by which all investigators could evaluate and sort the statements into concepts. The content analysis was conducted via a card sorting procedure. The first stage was to separate the generic HrQoL statements from specific growth related HrQoL statements, the specific statements were sorted into conceptually similar areas and thematically labeled thus providing collections of statements reflecting the same theme in different languages. This process then was cross-checked by other groups so that the representativeness of the categories found was validated within the process. The statements were sorted into the dimensional models and sub-domains were used as guidelines for the inclusion of items that were relevant in terms of content. Each statement sub-domain was discussed in the expert group to determine the core statements which uniquely reflected the concepts. A decision was then made about the wording and structure of the response categories so that a self-reported, pen and paper questionnaire for children 8 to 18 years of age was available for pilot testing. In addition, the conceptual model developed as a result of the focus group categorization process led to the differentiation between HrQoL domains and their potential determinants.

The pilot questionnaire was assembled in English, and items as well as the response scales were back-translated into the respective languages by native speakers bilingual in English. The translated version was reconciled with the phrasing of the original focus group items generated in each country. Back-translations were conducted according to international standard translation procedures [46].

### Pilot testing

All previous focus group participants and subjects previously contacted but unable to attend the focus groups were invited to the centers to participate in the initial pilot test and cognitive debriefing exercise. Each country included at least 24 children or adolescents but was free to include additional subjects to ensure that a large enough sample was available. The pilot test questionnaire data were complemented by clinical, socio-demographic and psychosocial data of the young patients and their parents. A cognitive debriefing exercise followed the completion of the instrument by the participant families. In the cognitive debriefing, children and adolescents were asked to rate a subset of items regarding clarity and relevance for young persons with dSS, importance to the individual situation and if rewording could improve the item. A five-point, Likert-type response scale was used with response options of: not at all/never, slightly/seldom, moderately/quite often, very/very often, extremely/always. The understandability of these response scale categories was also assessed in the cognitive debriefing.

The results from the pilot test were analyzed with regard to preliminary operating characteristics and psychometric properties to guide further selection of items. Preliminary analysis results were reviewed together with the feedback from the cognitive debriefing, allowing the researchers to make decisions regarding item retention for the final field test version of the questionnaire.

### Field test

In the field test the QoLISSY questionnaire was administered to newly identified study participants meeting inclusion criteria. Additional measures were included in the field test to identify potential determinants and components of HrQoL as well as for construct validation purposes. Among these was the KIDSCREEN-52, a generic HrQoL measure to estimate convergent validity [45]. Subjects approached for and agreeing to the re-test were sent a second mailing about two weeks later with the aim to receive data from 50% of the sample to examine test- retest reliability.

The operating characteristics and psychometric properties of the field test instrument were analyzed at the item and scale levels according to classical test theory [47]. Specifically, items were individually inspected for missing values, distribution characteristics (mean, SD, skewness and kurtosis) and item difficulty. Principal component factor analyses was conducted to explore the dimensionality of the core HrQoL domains and the additional determinant scales, as specified in the conceptual model derived from focus group results. On the scale level, analyses were conducted to evaluate the score distribution and the reliability of the scores (internal consistency, retest and split-half reliability in terms of intraclass correlation coefficients). Criterion validity of the QoLISSY instrument was tested via correlations with the generic KIDSCREEN instrument and via analysis of known group differences regarding diagnosis, treatment status and height.

Confirmatory factor analysis (CFA) was used to test the dimensional structure of the QoLISSY questionnaire. The models’ goodness of fit was assessed using the main fit indexes: maximum-likelihood χ2 p-value and χ2/degrees of freedom, comparative fit index (CFI) and root mean square error of approximation (RMSEA). The reference values of χ2/df ≤ 2, CFI ≥ .95 and RMSEA ≤ .06 were considered indicators of model´s good fit to the data; and the reference values of χ2/df ≤ 5, CFI ≥ .80 and RMSEA ≤ .10 were considered the threshold for assessing the model’s fit as acceptable. All statistical analysis analyses were performed using SPSS (IBM SPSS Statistics V 18) as well as with specific programmes which had been used in previous international HrQoL projects [48].

Results below are presented to provide information on the psychometric performance of the instrument and not to identify differences in populations or evaluate the impact of clinical characteristics on HrQoL, which would require a different study design.

## Results

The overall sample across phases of the instrument development consisted of 446 children. Eighty-four children were included in the focus groups and 94 children in the pilot test phase. In the field test, 268 children returned the questionnaire of which 124 additionally completed and returned the retest questionnaire.

The number of participants as well as diagnosis, treatment status, age group and gender varied across countries with each contributing sufficient numbers to ensure the representativeness of the sample in each country: 89 in France, 118 in Germany, 84 in Spain, 109 in Sweden and 48 in UK.

### Focus group results

A total of 84 patients, 38 children (8–12 years) and 46 adolescents (13–18 years), with ISS and GHD were recruited into 28 focus groups with an average 4 participants, specifically, the majority having been treated with GH (Table 1).

**Table 1:** Focus group and pilot test sample of children and adolescents according to age, diagnosis and treatment

**Age group**	**Diagnosis**	**Treatment status**	**Focus groups**	**Pilot test**
8-12 yrs	ISS	treated	6	8
		untreated	15	11
	GHD	treated	17	27
		untreated	0	4
13-18 yrs	ISS	treated	15	9
		untreated	13	18
	GHD	treated	17	16
		untreated	1	3
**Total**			84	96

ISS: idiopathic short stature; GHD: growth hormone deficiency.

**Figure 1:**
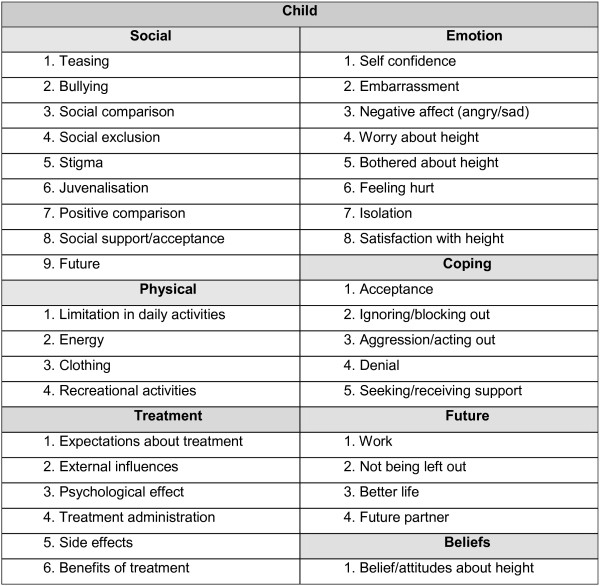
Domains and sub-domains resulting from the focus groups.

From the focus group discussions a total of over 4000 statements were identified and condensed. The statements were organized into domains and sub-domains (Figure 1). The statement list was reduced in an investigators consensus meeting by removing repetitious and ambiguous items. The most frequently expressed ideas consistent across countries were retained as well as frequently mentioned concepts within a country to ensure the greatest degree of content validity.

**Figure 2:**
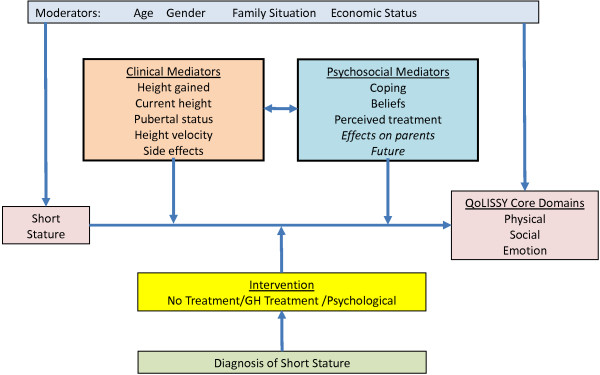
Conceptual model of the QoLISSY questionnaire.

The conceptual model specifies the three core domains of HrQoL, (physical, emotional and social) as well as potential mediators or determinants in terms of coping, beliefs, treatment and concerns regarding the future. In addition, moderator variables such as age, gender, family situation and socio-economic status were included. This model conceptualizes HrQoL as an outcome or dependent variable, which is related to the child’s stature as an independent variable on the background moderators and describes how growth related HrQoL is affected by clinical and psychosocial mediators regulating the intensity of impact of dSS on HrQoL (Figure 2).

Following the statement reduction process a total of 124 items were formulated and arranged into 7 scales for child report. Individual items were constructed to include both positive and negative wording though the predominant direction describes impairment. Scale level scores were constructed based on the mean item score for the particular scale and standardized on a scale of 0 to 100 such that higher scores reflected better quality of life.

### Pilot test results

The pilot test was conducted on a convenience sample of 94 children (48 children and 46 adolescents across the European centers) and reflected the intended range of age, gender and diagnosis (refer to Table 1).

Some items were discarded at this stage because participants judged them negatively during the cognitive debriefing, e.g. the items were not applicable for the participants or were not easily understood. This information was combined with the results of the item analyses and preliminary psychometric testing. Using descriptive and psychometric indicators, the behaviour of items and domains or scales was assessed and poorly performing items were removed. This process was conducted in a second consensus discussion, resulting in a refined list of 53 candidate items across six of the originally seven scales. The Future scale was removed as a component of the children’s questionnaire and included as a domain in the parent version (see Table 2).

**Table 2:** Results of cognitive debriefing

	**No. of items**	**Min/max**	**Mean**	**SD**	**Skewness**	**Kurtosis**	**Relevance**	**Clarity**	**Importance**	**Re-worded**	**Items rejected**	**Items moved between domains**	**Items retained**
							**%**	**%**	**%**	**%**	**N**	**N**	**N**
Physical	10	1-5	2.0	1.1	1.1	0.7	82.5	94.6	75.6	9.2	2	−1	7
Social	20	1-5	2.3	1.0	0.8	2.5	85.9	96.0	82.8	11.5	13	−1/+2	8
Future	6	1-5	2.0	1.1	1.1	1.1	82.2	96.9	77.8	7.8	2	4*	___
Emotion	16	1-5	2.2	1.1	0.9	0.7	88.3	97.6	86,6	13.8	7	−2/+2	9
Coping	22	1-5	2.5	1.2	0.5	−0.2	89.1	96.0	86,2	7.9	11	−1	10
Beliefs	16	1-5	2.0	1.2	1.0	0.3	84.6	97.4	73.1	6.3	12	0	4
Treatment	34	1-5	2.4	1.2	0.7	1.1	86.7	96.1	85.3	5.8	19	0	15
Total	124												53

Scale descriptive data (first 6 columns), respondent feedback (%) on relevance, clarity, importance and need to reword items (next 4 columns), number of items rejected and moved (next 2 columns); number of items retained for field test (last column). Child and parent feedback combined (n=98 – 190) Domain “Future” was moved to parent version.

### Field test

A previously identified convenience sample of parents with children meeting study inclusion criteria were mailed the instrument. A total of 544 questionnaires were sent out to families and 337 were returned to the growth clinics. The return rate across countries ranged from 48% to 92%. Subtracting empty or incomplete questionnaires as well as accounting for the fact that only parents but not their children had responded, 268 returned patient questionnaires were included in the analysis. (n=129: 8–12 years; n=139: 12–18 years, Table 3). In addition, 124 retest responses were received, representing about 50% of the sample as planned. Inspection of item performance resulted in discarding three items from the child version, so that the field test version included 50 items. The results were evaluated for reliability and validity, including criterion and construct validity.

**Table 3:** Sociodemographic and clinical characteristics of children and adolescents in the field test sample by age group

**Subject characteristics**		**Total**		**Age group**		**Age group**	
				**8**–**12 yrs**		**13**–**18 yrs**	
N		268	100%	129	48.1%	139	51.9%
Gender	Female	114	42.5%	60	52.6%	54	47.4%
	Male	154	57.5%	69	44,8%	85	55,2%
Main diagnosis	GHD	109	40.7%	40	36.7%	69	63.3%
	ISS	159	59.3%	89	56.0%	70	44,0%
Treatment status	Treated	142	53,0%	58	40,8%	84	59,2%
	Untreated	126	47,0%	71	56.3%	55	43.7%
Height SDS	0 to −1.499	77	31.4%	31	40.3%	46	59.7%
N = 246	−1.50 to −2.499	115	46,9%	67	58.3%	48	41.7%
	−2.50 and lower	53	21.6%	21	39.6%	32	60.4%

GHD: growth hormone deficiency; ISS: idiopathic short stature; SDS: standard deviation score.

Exploratory factor analysis was used both to identify the scale structure and to support the scoring system of the questionnaire. In line with the conceptual model, items representing the core domains (physical, social and emotional) were subject to a principal component factor analysis, with three factors extracted. The first factor with an Eigenvalue of 10.62 explained 48.3% of the variance, the second added 6.0% to the variance with an Eigenvalue of 1.33 and the third another 4.6% with an Eigenvalue of 1.03, all three contributed to a cumulative 59% of variance explained (full analysis for all items not shown here). Conceptually, the three core scales formed the QoLISSY-QoL total score, with the three domains (Coping, height-related Beliefs, Treatment) serving as additional determinant modules.

The scale distribution characteristics from the overall dataset suggest a relatively high level of HrQoL in the respondents with mean scores in the low 70’s for the core domains and total QoLISSY-QoL. The magnitude of the variance (SD) indicates good variability across the scales, and the low floor and ceiling effects as well as acceptable skewness and kurtosis suggest appropriate operating characteristics (Table 4).

**Table 4:** Psychometric properties of the QoLISSY child self reported scale in the field test sample

**Domain**	**Descriptive statistics**							**Reliability**				
	**N Items**	**N Children**	**Mean**	**SD**	**Skewness**	**% Floor**	**% Ceiling**	**N**	α	**Split-half**	**N**	**ICC**
**Physical**	6	268	73.69	22.80	-.951	0.4	12.3	263	.84	.83	120	.80
**Social**	8	268	72.94	22.93	-.787	0.4	10.4	265	.87	.83	121	.80
**Emotional**	8	268	72.69	23.87	-.938	1.1	10.1	259	.88	.88	121	.85
**Coping**	10	257	55.60	22.38	-.190	1.2	1.2	244	.82	.65	110	.56
**Beliefs**	4	266	69.13	28.59	-.776	3.8	21.1	263	.85	.85	117	.83
**Treatment**	14	152	55.12	21.06	-.125	0.7	0.7	143	.87	.74	72	.73
**QoLISSY total score**	22	268	73.10	21.39	-.802	0.4	4.9	251	.95	.92	120	.88

α : Cronbachs Alpha; ICC: intra class correlation coefficient.

N for treatment scale is low because domain was answered only by GH- treated patients; QoLISSY total score is sum of Physical, Social and Emotional domains.

In terms of internal consistency, Cronbach’s α reliability was 0.8 and above, with less than 10 items per scale. Split-half reliability was slightly lower but still satisfactory psychometrically (>.70) [47]. The test-retest reliability, calculated from the retest of a sample of 124 children and adolescents in terms of the intraclass correlation coefficient (ICC, last column in Table 4) was satisfactory with the exception of the Coping scale.

Construct validity was assessed by confirmatory factor analysis of the three core QoL scales and was subsequently repeated for the three mediator domains (not reported here).

**Figure 3:**
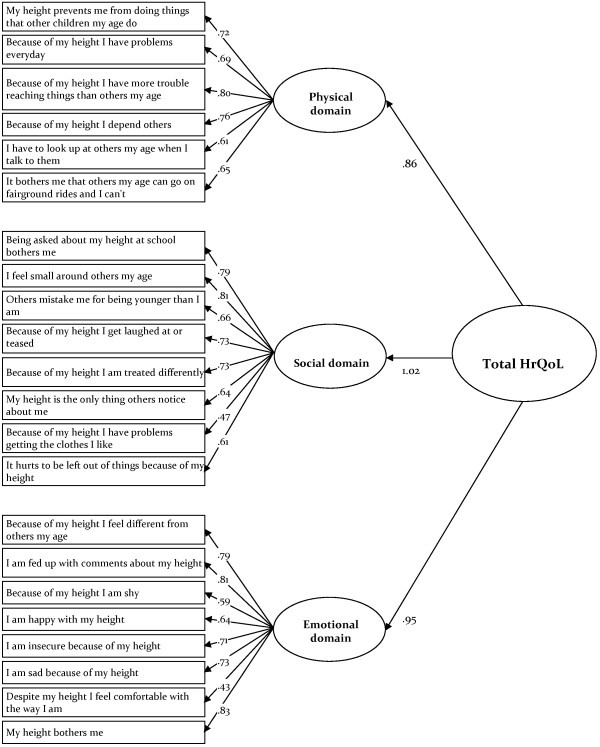
Three domain core HrQoL model (Child and Adolescent self report).

The factorial model of the patient-reported QoLISSY core instrument, which comprises the physical, social and emotional domain and a total score of HrQoL, had an acceptable fit, with Χ2 (206, n = 263) = 615.35, p <.05, Χ2/df = 2.99, CFI = .88 and RMSEA = .087. All items showed factorial validity, with significant factor loadings (p < .001) and, except for one item of the social domain and of the emotional domain, standardized regression weights were above the threshold of .50 (Figure 3).

The QoLISSY instrument showed high inter-correlations of the three cores QoL scales while the association with the Coping subscale was lower (Table 5).

**Table 5:** QoLISSY child self reported scale score intercorrelations (Person r)

	**Physical**	**Social**	**Emotional**	**Coping**	**Beliefs**	**Treatment**
**Physical**						
**Social**	.788**					
**Emotional**	.709**	.830**				
**Coping**	-.064	-.025	-.002			
**Beliefs**	.566**	.556**	.635**	.023		
**Treatment**	.116	.050	.065	.280**	-.141	
**QoLISSY total score**	.901**	.946**	.920**	-.032	.638**	.084

**. Correlation coefficient is significant at the 0.01 level.

QoLISSY total score is sum of Physical, Social and Emotional domains.

Criterion validity was examined in terms of convergent validity using a generic measure and in terms of known groups validity using participants clinical data.

The QoLISSY scale scores demonstrated significant, but only moderate, correlations with the physical and psychological well-being dimensions of the KIDSCREEN-52. These correlations support thematic agreement, but also suggest that the two instruments may not be measuring identical constructs (Table 6).

**Table 6:** Correlation coefficients (Pearson r) for the QoLISSY child self-report scales with subscales of KIDSCREEN-52 child version

		**KIDSCREEN-52**									
		**Physical well-being**	**Psychological well-being**	**Mood**	**Self**	**Autonomy**	**Parents**	**Financial**	**Social**	**Schooling**	**Bullying**
**QoLISSY**	**Physical**	.20**	.25**	.33**	.50**	.25**	.25**	.30**	.23**	.21**	.40**
	**Social**	.18**	.29**	.38**	.51**	.29**	.29**	.28**	.28**	.24**	.51**
	**Emotional**	.25*	.37**	.41**	.58**	.34**	.34**	.30**	.29**	.29**	.43**
	**Coping**	.16*	.20**	.09	.15*	.16*	.17**	.11	.23**	.18**	.10
	**Beliefs**	.22**	.31**	.41**	.47**	.33**	.31**	.22**	.24**	.21**	.32**
	**Treatment**	.23**	.20*	-.01	.24**	.11	.14	.08	.16*	.12	.08
	**QoLISSY total score**	.23**	.33**	.41**	.57**	.32**	.32**	.32**	.29**	.27**	.48**

Correlation Coefficients, *Significant at p<0.05, **Significant at p<0.01.

QoLISSY total score is sum of Physical, Social and Emotional domains.

In terms of known group validity, significant differences were found between ISS and GHD patients (Table 7) between untreated vs. GH-treated children (Table 8) and between degrees of SS (very short vs. less short children, Table 9). Extremely short patients (≤ −2.5 SD, n = 53) were characterized by significantly lower HrQoL than those in the mid range (−1.5 to −2.49 SD, n = 116) or the taller patients in the upper range (0 to −1.49 SD, n = 77).

**Table 7:** QoLISSY child self-reported scales score: means, standard deviations and group differences by diagnosis

**Diagnosis**							
	**ISS**		**GHD**		**t**	**df**	**p**
	**M**	**SD**	**M**	**SD**			
**Physical**	69.28	23.82	80.12	19.62	4.07	266	<.001
**Social**	67.57	23.17	80.77	20.26	4.94	266	<.001
**Emotional**	68.37	24.75	78.98	21.08	3.66	266	<.001
**Coping**	55.20	21.47	56.21	23.78	.352	255	.725
**Beliefs**	63.71	28.56	76.95	26.88	3.81	264	<.001
**Treatment**	56.36	21.84	54.42	20.70	-.544	150	.587
**QoLISSY total score**	68.41	21.93	79.96	18.66	4.50	266	<.001

ISS: idiopathic short stature; GHD: growth hormone deficiency;

M: mean; SD, standard deviation; t: t-value; df: degrees of freedom.

QoLISSY total score is sum of Physical, Social and Emotional domains.

**Table 8:** QoLISSY child self-reported scales scores: means, standard deviations and differences in by growth hormone treatment status

**Treatment status**							
	**Treated**		**Untreated**		**t**	**df**	**p**
	**M**	**SD**	**M**	**SD**			
**Physical**	80.15	19.60	66.42	24.01	5.09	266	<.001
**Social**	80.14	20.63	64.82	22.76	5.74	266	<.001
**Emotional**	78.20	22.11	66.47	24.33	4.14	266	<.001
**Coping**	54.17	23.94	57.16	20.52	−1.07	255	.286
**Beliefs**	71.96	28.86	65.99	28.06	1.71	264	.089
**QoLISSY total score**	79.49	18.95	65.90	21.77	5.42	266	<.001

M: mean; SD: standard deviation; t: t-value; df: degrees of freedom.

QoLISSY total score is sum of Physical, Social and Emotional domains.

**Table 9:** QoLISSY child self reported scales score: means, standard deviations and differences by degree of short stature

**Degree of short stature (SDS height)**									
	**Upper group**		**Mid group**		**Lower group**		**F**	**df**	**p**
	**0 to −1.49 SD**		**1.5 to −2.49 SD**		**≤ −2.5 SD**				
	**M**	**SD**	**M**	**SD**	**M**	**SD**			
**Physical**	86.68	13.34	70.62	23.35	58.02	22.72	31.81	244	<.001
**Social**	85.45	17.08	68.75	23.12	59.37	20.25	27.34	244	<.001
**Emotional**	84.65	17.08	68.61	24.44	61.02	22.12	20.95	244	<.001
**Coping**	53.65	25.19	55.02	23.07	59.99	16.99	1.28	235	.279
**Beliefs**	76.92	26.48	68.08	29.51	59.08	27.70	6.29	242	.002
**Treatment**	58.50	18.46	49.96	24.43	54.91	17.88	2.32	134	.102
**QoLISSY total score**	85.59	13.90	69.33	21.67	59.47	19.60	32.04	244	<.001

M: mean; SD: standard deviation F: F-value; df: degrees of freedom; p: significance level.

QoLISSY total score is sum of Physical, Social and Emotional domains.

The operating characteristics and psychometric properties of the child version of the questionnaire in the field test thus fulfilled internal consistency, split half and retest reliability as well as content, construct and criterion validity criteria.

## Discussion

The current paper describes the development and testing of an instrument to assess the HrQoL of children and adolescents with dSS. By following guidelines for patient-reported outcome instrument development and in cooperation with clinical and methodological experts in five countries, a comprehensive, methodologically sound and practical instrument was developed, which can be used for clinical research as well as a tool to evaluate treatment benefits in a practice environment.

Conceptually, the development process was guided by the need to address the diverse subjective concerns of children and adolescents whose families seek consultation and treatment related to short stature. The use of focus groups across age ranges, diagnoses and countries was a prerequisite for assuring a broad level of input and content saturation. Through the focus groups, it was possible to identify the relevant concepts associated with HrQoL for both child and adolescent age groups, also taking into account gender, diagnosis, treatment status and degree of short stature across countries.

Statements from the focus groups were instrumental in identifying the relevant concepts for item generation and in guiding the development of the conceptual model. The cognitive debriefings added to the refinement of the items and provided evidence in support of the conceptual framework, particularly in the separation of concepts salient to children as compared to their parents. The preliminary evidence of content and construct validity from the pilot testing phase suggested that though nominal differences in performance and linguistic interpretations were detected, the conceptual framework, domains and items would show sufficient robustness across languages in the field and retest phase.

It is noteworthy that the development of the QoLISSY questionnaire was a simultaneous process conducted concurrently in five European countries. This aspect is rather innovative, as is the targeted nature of the instrument that focuses on both GHD and ISS, is available for patients self report from 8 to 18 years of age (and, as will be described in a separate paper, for parents) and specifies not only indicators but also determinants of HrQoL. All of this makes QoLISSY unique and differentiates it from available HrQoL measures in short stature youth.

Although overall the acceptance of the instrument and its psychometric performance during the development process was positive, some limitations must be considered. A challenge of this study was to recruit across countries a comparable number of subject families with equivalent clinical characteristics, especially as it related to diagnosis and treatment. Furthermore, responsiveness of the instrument to differences in HrQoL according to treatment cannot be inferred from the cross-sectional data reported here, so that additional longitudinal research is needed.

In Europe, children with ISS are rarely treated with GH so that this group was underrepresented. Respondent fatigue, particularly in the youngest cohort, was a concern although anecdotal evidence suggests that it was not a substantive problem. In addition, the children were asked to answer a number of in-depths questions regarding their height - related functioning and well-being, especially those participating focus groups and in the pilot with cognitive debriefing sessions. In the field test, additional HrQoL instruments were included to allow for construct validation, which increased the burden for respondents even though the field test version of the QoLISSY instrument is reasonably short.

The focus of the questionnaire is on the HrQoL impact of short stature as an isolated physical characteristic in a population seeking health-care. The intended use of the questionnaire is within a medically defined population which will allow interpretation of the results related to clinical and psychosocial mediators as described in the conceptual model included in Figure 2. This model illustrates the complex nature of HrQoL measurement in this area, in that a number of factors have to be taken into consideration in relation to HrQoL measurement.

The QoLISSY questionnaire provides one, previously missing, element in that investigation. The tabled results from this study are limited in their generalizability due to the nature of the sample, though the instrument could be useful to assess the height impact on HrQoL in other diagnoses. Further studies will be needed to identify differences in levels of well-being and functioning according to treatment status, pubertal status, age and gender. The current dataset provides a valuable opportunity to examine the contribution of psychosocial determinants such as coping mechanisms, social support, behavioural problems and parents’ own well-being to child-reported HrQoL. In addition, cross cultural performance of the instrument in terms of its equivalence across language versions needs to be examined.

The European QoLISSY study is an example of international cooperation in the development of patient-reported outcomes in a rare but important health condition impacting on the lives of children and their families. Currently, the QoLISSY instrument is available in five languages and additional validation work for other languages and environments is ongoing. Access to the instrument is provided through a manual available at production costs [49].

Hopefully, this tool will also be used in descriptive HrQoL studies to identify patient needs for care. Patient needs derived from HrQoL screening may suggest treatments for SS that do not only include GH substitution but also psychological interventions. If applied in clinical outcomes research, the QoLISSY instrument might also be used to comparatively quantify the benefit of such therapies in health economic evaluation and to monitor their implementation in standard care.

## Abbreviations

Df: Degrees of freedom; DIF: Differential item functioning; GH: Growth hormone; GHD: Growth hormone deficiency; HrQoL: Health-related quality of life; ICC: Intra class correlation; IRT: Item response theory; ISS: Idiopathic short stature; M: Means; QoLISSY: Quality of Life in Short Stature Youth; SD: Standard deviation; SDS: Standard deviation score; SS: Short stature; T: t-test statistic

## Competing interests

The project was funded by Pfizer.Ltd and the authors were provided funding for its conduct at each site. There are no additional competing interests to report.

## Authors’ contributions

MB was the coordinating principle investigator and provided direct input into the design and execution of the study and the preparation and review of the manuscript. JQ provided direct oversight of the study conduct, conducted child and parent interviews and debriefs, organized the qualitative and quantitative content analysis, and contributed to the preparation and review of the manuscript. JC, MH, MP, EM, and EF where the principle investigators in Sweden (JC), Spain (MH), United Kingdom (MP) and France (EM and EF) respectively, coordinated and administered the study including the focus group interviews, pilot test and cognitive debriefings and field with re- test, participated in the item content analysis and instrument design, and contributed to the preparation and review of the manuscript. KD, KL, MD, DS, and AR provided operational support and participated in the interview processes in the UK (KD), Sweden (CL and MD), Spain (DS) and Germany (AR) respectively, participated in the item content analysis and instrument design, and reviewed the final manuscript. AP and HW provided oversight of the project and participated in the item content analysis and instrument design, execution of the field test, and contributed to the preparation and review of the final manuscript. MP managed the data entry, psychometric analysis, and statistical aspects of the study (with KD), and contributed to the preparation and review of the final manuscript. All authors read and approved the final manuscript.
